# Amoebic liver abscess: diagnosis and management of a single case in Guangdong, China

**DOI:** 10.1186/s12879-025-10623-0

**Published:** 2025-03-12

**Authors:** Yixin He, Dana Huang, Ying Yang

**Affiliations:** 1 Department of Emergency, Peking University Shenzhen Hospital, Shenzhen, Guangdong, China; 2 Shenzhen Center for Disease Control and Prevention, Shenzhen, Guangdong, China

**Keywords:** Entamoeba histolytica, Amoebic liver abscess, Charcot-Leiden crystals, Liver puncture drainage

## Abstract

**Background:**

Amoebic liver abscess is a hepatic infection due to the invasion of Entamoeba histolytica. This parasitic infection is globally distributed, particularly in tropical and subtropical regions. A remarkable decline in the incidence of amoebic liver abscesses has been observed in recent years thanks to economic and public health progress.

**Case presentation:**

This case report describes the symptoms of a 32-year-old male with an amoebic liver abscess. Despite the diagnostic difficulties encountered, favorable outcomes were obtained after the appropriate drainage of the abscess and the appropriate anti-amoebic therapy by the administration of metronidazole.

**Conclusions:**

The management of amoebic liver abscess requires a meticulous evaluation of the patient’s clinical manifestations, the severity of the condition, and the response to therapeutic interventions. Early identification, combined with timely and effective treatment, is essential to reduce the risk of complications and the mortality rate.

**Clinical trial number:**

Not applicable.

## Background

Amoebiasis is an infection caused by the protozoan parasite Entamoeba histolytica, with lesions commonly occurring in the colon. The pathogen can further migrate to the liver, lungs, and brain, although in a minority of cases [1]. This pathogen is widespread worldwide, with a higher incidence in tropical and subtropical regions [2]. Amoebic liver abscess (ALA) is a common extraintestinal manifestation of amoebiasis, with a higher prevalence in adult males, approximately 7–10 times higher, compared to other demographics. The disease most commonly affects adults aged 20–40 years [3]. The clinical presentation of ALA is influenced by its course, size and location of the abscess, as well as the complications. Misdiagnosis or missed diagnosis is common due to its complex clinical manifestations and atypical early symptoms [4]. Despite the decline in the incidence of ALA due to improved living standards, it is essential to maintain vigilance in clinical practice. Therefore, this work reports a case with a detailed description of how the diagnosis was achieved as well as the treatment of a patient with ALA.

## Case presentation

A 32-year-old male patient was admitted to the Peking University Shenzhen Hospital on March 15, 2024, suffering from fever for five days and reaching a maximum temperature of 40.0 ℃. The patient reported no abdominal pain, diarrhea, nausea, vomiting, or jaundice. The patient initially was subjected to an abdominal CT scan at another medical facility, revealing a low-density lesion in the S7/8 hepatic segment, raising suspicion of the presence of a liver abscess. However, no treatment was administered at that time. Despite the self-administration of ibuprofen, acetaminophen, and cephalosporin antibiotics, the patient’s fever persisted, prompting a visit to our emergency department. The patient had a history of uveitis in both eyes 2 years ago for which he received steroid treatment for six months. He did not have any history of hepatitis, travel to endemic regions, amoebic dysentery, or familial infections. He also had no history of homosexuality but admitted to having had heterosexual intercourse more than a month earlier and subsequently self-administered an oral HIV prophylaxis medication for 2 days. The patient had a documented history of smoking and alcohol consumption.

The patient’s physical examination at admission revealed the following features: height of 175 cm, weight of 70.5 Kg, blood pressure of 121/69 mmHg, pulse rate of 111 beats per minute, and temperature of 38 ℃. The cardiopulmonary examination did not show any abnormality. The patient’s abdomen was noticeably tender to palpation, with tenderness in the liver region and a negative Murphy’s sign. Clinical laboratory tests revealed high inflammatory markers, liver dysfunction, and abnormalities in coagulation function (Table 1). The patient was negative for hepatitis B surface antigen, antibodies against hepatitis C virus, antibodies against human immunodeficiency virus, specific antibodies against syphilis and tumor markers.

**Fig. 1:**
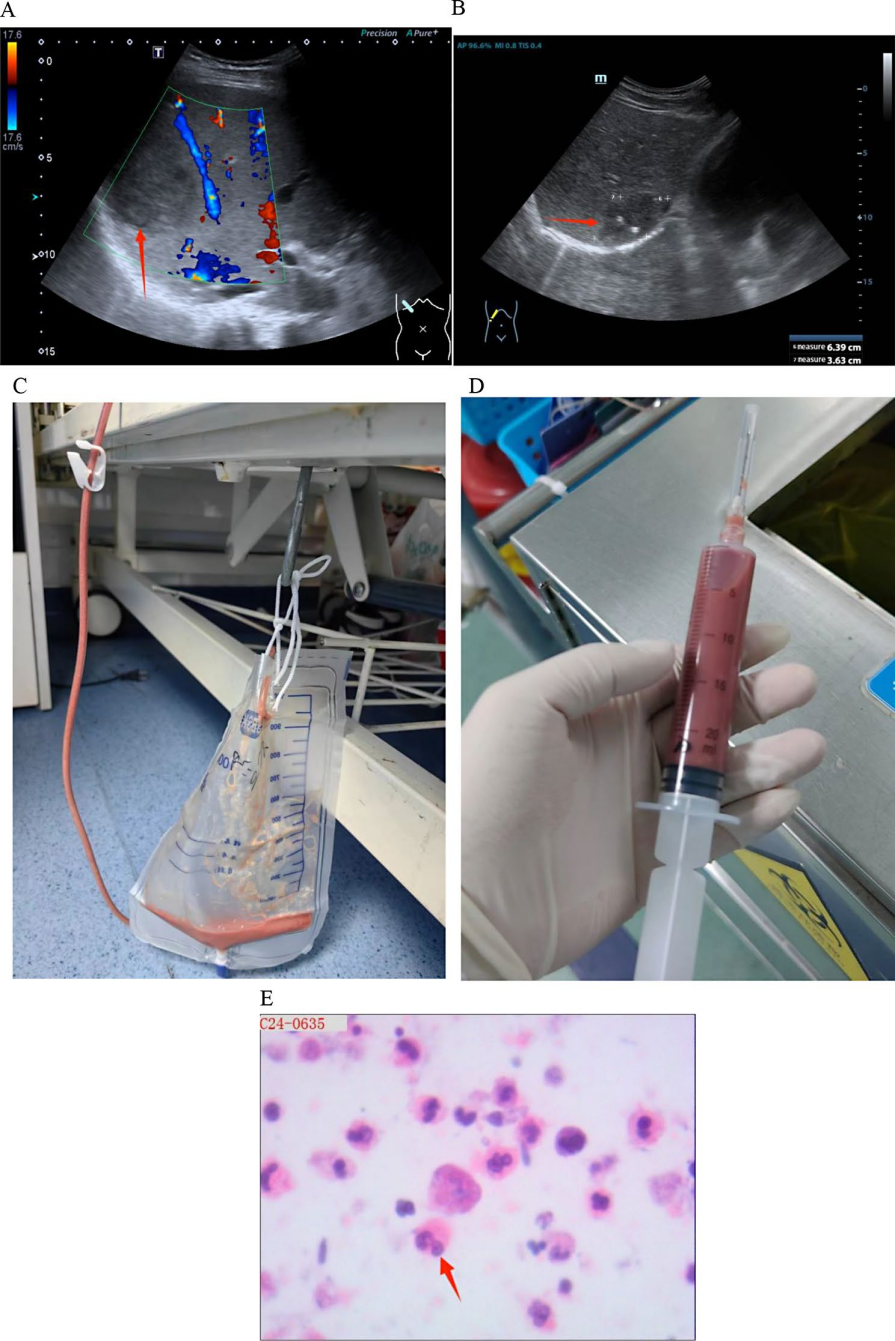
(Perfluorohexane) Liver ultrasound contrast on day 3 after admission (**A**). Re-examination of liver ultrasound on day 5 after the procedure (**B**). A pale pink, odorless and viscous fluid was collected on day 1 after the percutaneous catheter drainage of liver abscess (**C and D**). Histopathological analysis of the pus revealing an increased presence of granulocytes, histiocytes, and fragmented erythrocytes, along with necrotic-like material, consistent with abscess formation; no amoebic pathogens were identified (**E**)

**Fig. 2:**
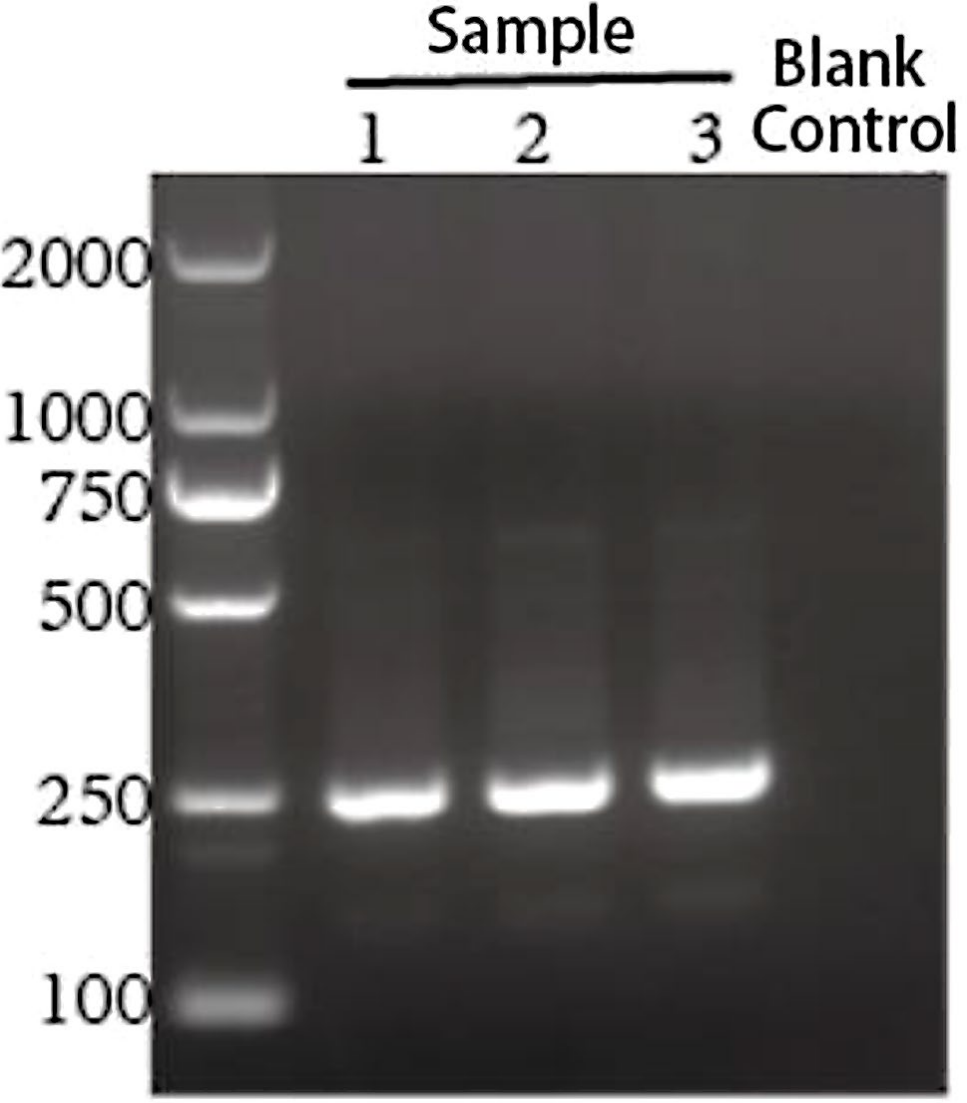
Nested PCR product gel electrophoresis results.Further amplification of the amoebic SSU rDNA gene was conducted using a nested PCR approach. The products from the second round of amplification were subjected to agarose gel electrophoresis. The results revealed that the test sample yielded a band of the expected size (246 bp) corresponding to the theoretical product, and the sequencing analysis confirmed that the amplified product matched the sequence of Entamoeba histolytica

**Fig. 3:**
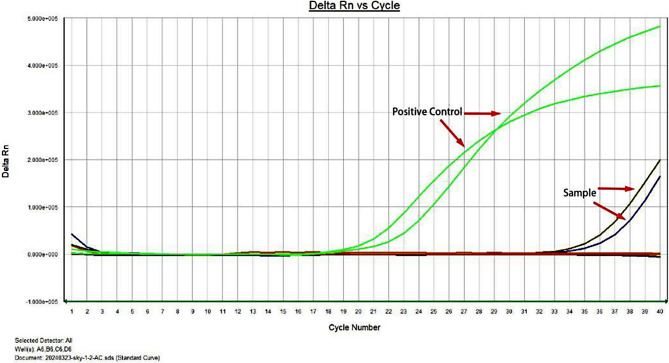
Nucleic acid fluorescence PCR results of patient abscess puncture fluid.Fluorescence PCR results showed that the threshold cycle (CT) value of the sample was about 34.5, and there was an obvious exponential increase. According to the results of the kit, it could be preliminarily interpreted as positive

On day 3 after admission, (sulfur hexafluoride) contrast-enhanced ultrasound examination of the liver showed a mixed mass (67 × 54 × 55 mm) at the junction of S7/8, with a quasi-round shape and a blurred boundary (Fig. 1A). The peripheral ring of the mass was low-enhanced after the injection of the SonoVue contrast agent, and the inner area was not enhanced, which was considered as the liquefied part of the liver abscess in the sonogram. Thus, ertapenem with levofloxacin was administered as empirical anti-infective therapy. Ultrasound-guided puncture and drainage of liver abscess was performed on day 5 after admission to further confirm the diagnosis, and 80 ml light pink and odorless viscous fluid was collected on the first day after operation (Fig. 1C and D). The morphological examination of the pus cells revealed that the smears were full of nucleated cells, mainly neutrophils, eosinophils, Charcot-Leiden crystals and orange blood crystals; routine bacterial and fungal culture were negative, and the patient refused to be subjected to mNGS test to confirm the result of the culture. Therefore, the results of the blood test showing increased eosinophils and the cytological results of pus supported the possibility of an amebic liver abscess. Left-oxyfloxacin was stopped on day 2 after surgery, and metronidazole 0.5 g q8h was administered for the treatment of an empiric microbial infection. However, samples of pus and stool were examined for trophozoites and Amoeba antigens, both resulting negative (Fig. 1E). The presence of a potential amoebic infection was further investigated by collecting the abscess fluid and urgently dispatching it to the Shenzhen Center for Disease Control and Prevention for analysis on day 2 after surgery. The test results revealed that entamoeba histolytica smear was negative by microscopy but positive by nested PCR (Fig. 2). Subsequent Sanger sequencing of PCR products confirmed a 99% match with the rRNA gene of entamoeba histolytica small subunit (serial number: X56991) along with positive fluorescence PCR results (Fig. 3). Considering both PCR sequencing outcomes and clinical symptoms, a diagnosis of entamoeba liver abscess was established. The follow-up liver ultrasound on postoperative day 5 after the drainage of the abscess and antibiotic therapy revealed a significant reduction in the abscess size to 64 × 36 × 31 mm compared to the previous measurement (Fig. 1B). Follow-up on postoperative day 11 revealed that the patient’s symptoms improved, and he was discharged with a drainage tube in place; metronidazole was taken orally out of the hospital. During the subsequent follow-up period, the patient underwent regular consultations at an external facility, with no recurrence of fever, and the liver abscess drainage tube was removed in mid-May 2024.

**Table 1:** Laboratory test results

Laboratory test	
WBC (×10^9^ /L)	20.42
NEU# (×10^9^ /L)	15.38
EOS# (×10^9^ /L)	1.70
PCT (ng/mL)	0.88
IL-6 (pg/mL)	215
TBIL (µmol/L)	36.5
ALP (U/L)	186
GGT (U/L)	115
PT (s)	15.8
APTT (s)	53.9

*WBC*: white blood cells; *NEU#*; neutrophil absolute count; *EOS*#: eosinophil absolute count; *PCT*: procalcitonin; *IL-6*: interleukin-6; *TBIL*: total bilirubin; *ALP*: alkaline phosphatase; *GGT*: gamma-glutamyl transferase; *PT*: prothrombin time; *APTT*: activated partial thromboplastin time

## Discussion

Amoeba liver abscess is caused by Entamoeba histolytica trophozoites entering the liver through blood flow, resulting in local liquefactive necrosis and abscess formation [5]. The abscess is usually located in the right lobe of the liver [6]. In this patient, the abscess occurred at the junction of S7 and S8 of the liver, which might be due to the fact that the right lobe is larger and has more blood supply than the left lobe and caudate lobe of the liver. The development of ALA typically occurs several weeks to several years after the initial amoebic infection [7], often due to a decline in the body’s immune system. The primary mode of amoeba transmission is through the ingestion of food or water contaminated with amoebic cysts and the amoeba is usually reaching the liver through the bloodstream, resulting in hepatic amoebiasis. Amoebic transmission may also occur through sexual contact or contaminated objects, although less frequent [8]. The patient had a history of sexual activity with the opposite sex over a month before the symptoms, but explained that the partner did not have similar symptoms.

The diagnosis of ALA is usually based on clinical manifestation, laboratory examination, imaging examination and etiological examination [9]. In terms of clinical manifestations, the onset of amoeba liver abscess is mostly slow, and the common symptoms include irregular fever, night sweats, loss of appetite, nausea, vomiting, abdominal distension, diarrhea, liver pain and weight loss [10]. ALA is usually a complication of amoebic colitis [11], but it can also occur independently [12]. This patient refused to undergo a colonoscopy examination, which made it impossible to determine the condition of the intestinal mucosa. However, the patient did not present any evident gastrointestinal symptom such as abdominal pain, diarrhea, nausea or vomiting. Additionally, stool pathogen testing did not reveal signs of amoebic infection. Laboratory examinations for ALA patients may reveal increased white blood cell counts, particularly neutrophils [13]. Serological tests such as the detection of amoebic antibodies may contribute to the diagnosis of the disease. The blood routine test of this patient indicated a significant increase in the absolute values of neutrophils and eosinophil. However, unfortunately, it was not possible to complete the serological testing for amoebic antibodies. In terms of pathogen examination, the diagnosis of ALA can be confirmed by obtaining the typical pus by liver puncture and finding amoebic trophozoites in the pus. The color and texture of the pus (usually brown or chocolate-colored, sometimes with a bloody odor) also contribute to the diagnosis [14]. The patient’s pus smear indicated the presence of eosinophils and Charcot-Leyden crystals, which supported our diagnosis and treatment. The literature review indicates that Charcot-Leyden crystals are formed as a product of the rupture of eosinophils containing Galectin-10 crystals, commonly seen in amoebic dysentery and allergic enteritis. Therefore, ALA was initially considered and the treatment plan was adjusted accordingly. However, the specific pathogen was further identified by sending the pus for testing to the Shenzhen Center for Disease Control and Prevention. The microscopic examination of the Entamoeba histolytica smear resulted negative.However, the nested PCR gave a positive result. Further analysis through Sanger sequencing of the PCR amplicons demonstrated a 99% homology with the small subunit ribosomal RNA gene of Entamoeba histolytica (accession number: X56991). Concurrently, the fluorescence-based PCR also gave a positive result. In conjunction with the PCR sequencing data and the patient’s clinical presentation, a definitive diagnosis of Entamoeba histolytica-induced liver abscess was made. Nevertheless, molecular diagnostic methods have certain limitations, such as the potential impact of sample contamination and the need for specialized laboratory equipment and trained personnel. Despite these challenges, molecular diagnostics continues to be one of the most effective tools in the diagnosis of amebic liver abscess to date [15].

The treatment of ALA mainly includes medical and surgical approaches. Medical treatment involves the use of anti-amoebic drugs and liver puncture drainage [16]. Commonly used drugs for treating ALA include metronidazole, tinidazole, and paromomycin [17]. These drugs work by killing the trophozoites of Entamoeba histolytica to treat the liver abscess [18]. In cases of mixed infection, appropriate antibiotics are selected based on the type of bacteria present. However, in this case, a satisfactory result was obtained using a combination of medical treatment with pus drainage, as evidenced by a significant reduction in lesion size during the follow-up after treatment by liver ultrasound. The prognosis of ALA usually depends on multiple factors, including early diagnosis, appropriate treatment, overall health condition of the patient, and the presence of complications. The timely and proper treatment results in a good prognosis in most patients. The limitations in the diagnosis and treatment of this patient include the failure to understand the cause of ALA in the patient and the failure to perform a thorough colonoscopy examination to screen for any intestinal lesions.

## Data Availability

The authors stated that all the data and materials were true and available in the study.

## Abbreviations

CT: Computed tomography
